# 1301. Assessing Safety and Clinical Outcomes of Outpatient Parenteral Antimicrobial Program in Veterans

**DOI:** 10.1093/ofid/ofad500.1140

**Published:** 2023-11-27

**Authors:** William Rich, Lauren H Epstein, Nora T Oliver

**Affiliations:** Emory University, Atlanta, Georgia; Emory University School of Medicine, Atlanta, GA; Atlanta VA Medical Center, Decatur, Georgia

## Abstract

**Background:**

Outpatient parenteral antimicrobial therapy (OPAT) is administering intravenous antibiotics in the outpatient setting. Herein we wish to understand the Atlanta VAMC (AVAMC) home OPAT population, infection details, and risk factors for hospital readmission, adverse drug events (ADEs), and clinical failure.

**Methods:**

Medical records of AVAMC veterans receiving home OPAT (not hemodialysis or infusion center) January 1, 2019 and June 30, 2022 were reviewed. Data collected included: demographic and co-morbidity data, OPAT indication/infection type, microbiological information, presence of surgical debridement, and antimicrobial selection and duration of therapy.

Outcomes included ADEs such as venous access events, laboratory abnormalities, and *Clostridioides difficile* infection. Other information include modification of OPAT plan (drug or duration), patient follow-up visit occurrence, re-hospitalization, and clinical resolution of infection. Univariate regression analyses were conducted on a subset of patients with musculoskeletal (MSK) infections (e.g. osteomyelitis, prosthetic joint infection).

**Results:**

A total of 149 veterans were reviewed, with median age 66 years, 92% male, and 44% white race. Median CCI score was 5, and 80/149 (54%) patients had diabetes. The majority of infections were bone infections (30%), followed by diabetic foot osteomyelitis (24%). Methicillin-sensitive *S. aureas* was the predominant bacteria isolated (20%). See included table for full details.

In the full cohort, 100/149(68%) had clinical resolution at 6 months and 20/149 (13%) had ADE during therapy. Rehospitalization occurred in 29 (20%) pts. Diabetes was associated with increased risk of failure -- Risk Ratio (RR) 1.68 (95% CI 1.01-2.77). Patients with clinic follow-up had a RR of 0.39 (95% CI: 0.21-0.73) for rehospitalization versus pts without follow-up.

For pts with diabetes in the MSK group (N=97), risk of failure was 1.50x higher than those without diabetes (95% CI: 0.67-3.34).The RR for rehospitalization was 0.23 (95% CI: 0.06-0.82) for patients who received follow-up compared to patients who did not receive follow-up.

**Figure:**
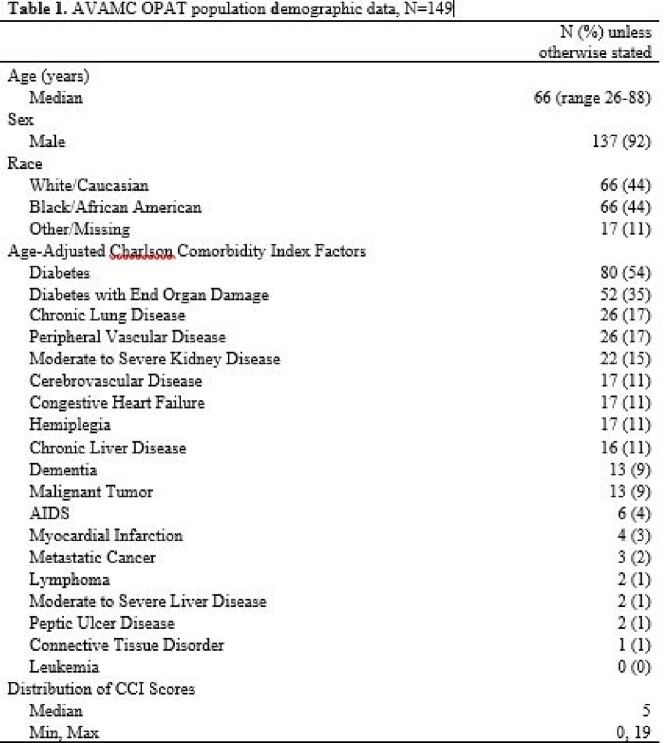

AVAMC OPAT population demographic data, N = 149

**Figure:**
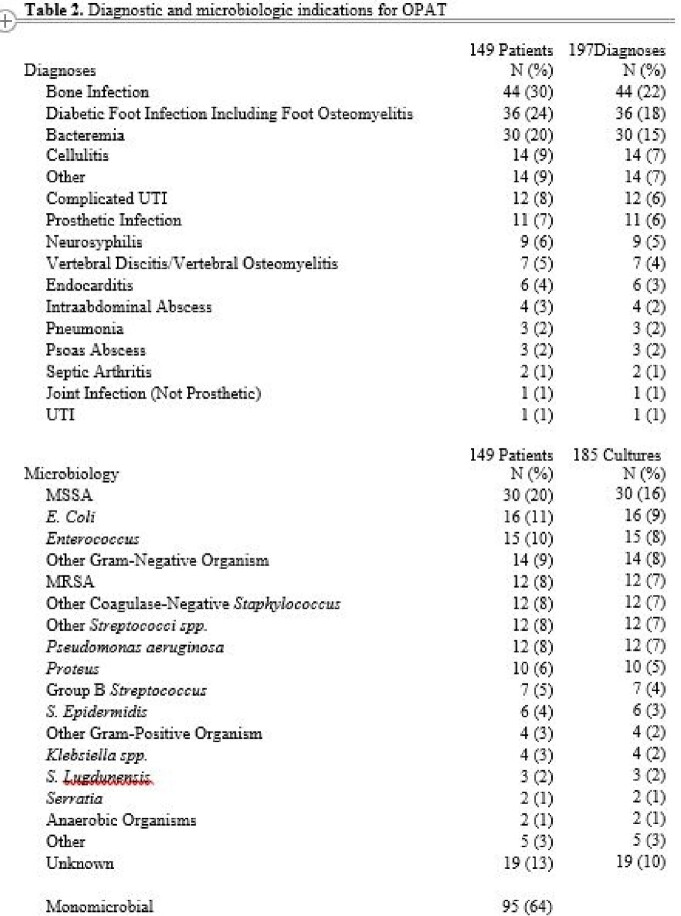

**Figure:**
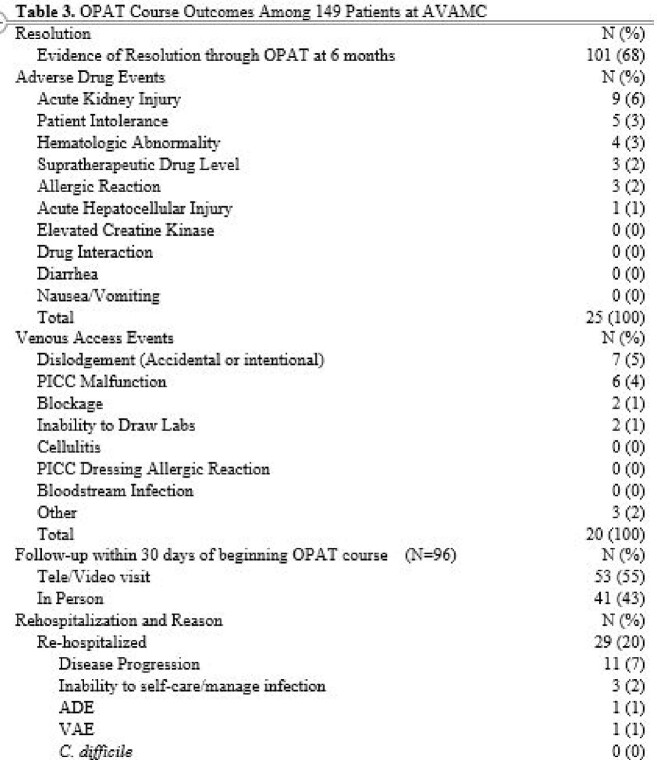

**Conclusion:**

The AVAMC OPAT cohort is an older group with largely MSK-related infections and MSSA. Few ADEs occurred, and clinical failure remained high.

**Disclosures:**

**All Authors**: No reported disclosures

